# Bis{μ-2-[1-(pyridin-2-yl­methyl­imino)­eth­yl]phenolato}bis­(azido­zinc)

**DOI:** 10.1107/S1600536811043455

**Published:** 2011-10-29

**Authors:** Jian-Ying Miao

**Affiliations:** a Department of Chemistry and Chemical Engineering, Baoji University of Arts and Sciences, Baoji 721007, People’s Republic of China

## Abstract

The title compound, [Zn_2_(C_14_H_13_N_2_O)_2_(N_3_)_2_], is a phenolate-bridged centrosymmetric dinuclear zinc(II) complex. The Zn⋯Zn distance is 3.076 (1) Å. Each Zn atom is five-coordinated by two O and two N atoms from two Schiff base ligands, and by one azide N atom, forming a square-pyramidal geometry.

## Related literature

For background on zinc complexes with Schiff base ligands, see: Keypour *et al.* (2010 ▶); Liu *et al.* (2011 ▶); You *et al.* (2011 ▶); Bhattacharjee *et al.* (2011 ▶); Das *et al.* (2010 ▶). For similar zinc complexes, see: Adams *et al.* (1995 ▶); You *et al.* (2009 ▶); Zhou *et al.* (2008 ▶); Basak *et al.* (2007 ▶).
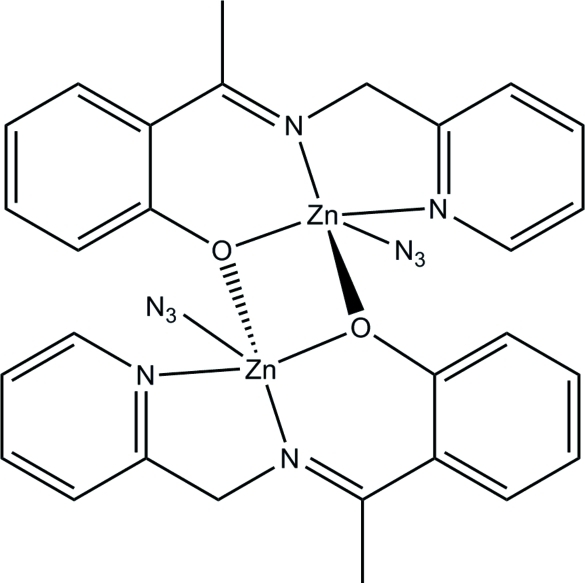


## Experimental

### 

#### Crystal data

[Zn_2_(C_14_H_13_N_2_O)_2_(N_3_)_2_]
*M* *_r_* = 665.33
Monoclinic, 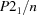

*a* = 10.057 (3) Å
*b* = 8.168 (3) Å
*c* = 16.741 (5) Å
β = 96.684 (3)°
*V* = 1365.8 (8) Å^3^
*Z* = 2
Mo *K*α radiation
μ = 1.80 mm^−1^
*T* = 298 K
0.23 × 0.23 × 0.22 mm

#### Data collection

Bruker SMART CCD area-detector diffractometer
Absorption correction: multi-scan (*SADABS*; Sheldrick, 1996 ▶) *T*_min_ = 0.682, *T*_max_ = 0.692
10859 measured reflections
2969 independent reflections
2254 reflections with *I* > 2σ(*I*)
*R*_int_ = 0.039

#### Refinement

*R*[*F* ^2^ > 2σ(*F* ^2^)] = 0.033
*wR*(*F* ^2^) = 0.074
*S* = 1.05
2969 reflections
191 parameters
H-atom parameters constrained
Δρ_max_ = 0.32 e Å^−3^
Δρ_min_ = −0.29 e Å^−3^

### 

Data collection: *SMART* (Bruker, 1998 ▶); cell refinement: *SAINT* (Bruker, 1998 ▶); data reduction: *SAINT*; program(s) used to solve structure: *SHELXS97* (Sheldrick, 2008 ▶); program(s) used to refine structure: *SHELXL97* (Sheldrick, 2008 ▶); molecular graphics: *SHELXTL* (Sheldrick, 2008 ▶); software used to prepare material for publication: *SHELXTL*.

## Supplementary Material

Crystal structure: contains datablock(s) global, I. DOI: 10.1107/S1600536811043455/qm2038sup1.cif

Structure factors: contains datablock(s) I. DOI: 10.1107/S1600536811043455/qm2038Isup2.hkl

Additional supplementary materials:  crystallographic information; 3D view; checkCIF report

## Figures and Tables

**Table 1:** Selected bond lengths (Å)

Zn1—N3	1.986 (2)
Zn1—O1^i^	2.0321 (17)
Zn1—O1	2.0733 (17)
Zn1—N1	2.106 (2)
Zn1—N2	2.107 (2)

Symmetry code: (i) 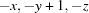.

**Fig. 1.**
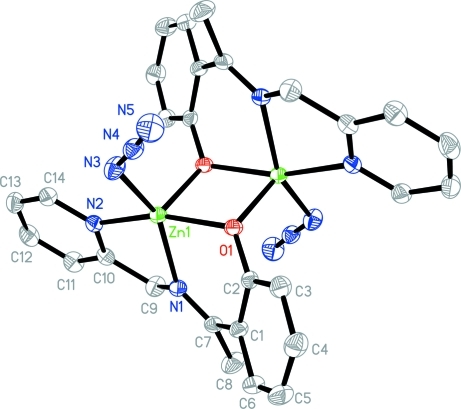
The structure of the title complex, showing 30% displacement ellipsoids (arbitrary spheres for the H atoms).

## supplementary crystallographic information

### Comment

Considerable attention has been focused on the zinc(II) complexes with multidentate Schiff base ligands (Keypour *et al.*, 2010; Liu *et al.*, 2011; You *et al.*, 2011; Bhattacharjee *et al.*, 2011; Das *et al.*, 2010). As an extension of the work on the structural characterization of such complexes, the title new dinuclear zinc(II) complex is reported here.

The title compound is a phenolato-bridged dinuclear zinc(II) complex, as shown in Fig. 1. The Zn···Zn distance is 3.076 (1) Å. Each Zn atom is five-coordinated by two O and two N atoms from two Schiff base ligands, and by one azido N atom, forming a square pyramidal geometry. The bond lengths in the square pyramidal coordination are comparable with those reported in similar zinc complexes with Schiff bases (Adams *et al.*, 1995; You *et al.*, 2009; Zhou *et al.*, 2008; Basak *et al.*, 2007).

### Experimental

2-Acetylphenol (1 mmol, 136 mg), 2-aminomethylpyridine (1 mmol, 108 mg), sodium azide (1 mmol, 65 mg), and Zn(NO_3_)_2_.6H_2_O (1 mmol, 297 mg) were dissolved in MeOH (80 ml). The mixture was stirred at room temperature for 1 h to give a colorless solution. The resulting solution was kept in air for a week, and block colorless crystals were formed.

### Refinement

H atoms were placed in idealized positions and constrained to ride on their parent atoms, with C—H distances in the range 0.93–0.97 Å, and with *U*_iso_(H) = 1.2 or 1.5*U*_eq_(C).

### Crystal data

**Table:**

[Zn_2_(C_14_H_13_N_2_O)_2_(N_3_)_2_]	*F*(000) = 680
*M**_r_* = 665.33	*D*_x_ = 1.618 Mg m^−^^3^
Monoclinic, *P*2_1_/*n*	Mo *K*α radiation, λ = 0.71073 Å
Hall symbol: -P 2yn	Cell parameters from 2133 reflections
*a* = 10.057 (3) Å	θ = 2.3–24.5°
*b* = 8.168 (3) Å	µ = 1.80 mm^−^^1^
*c* = 16.741 (5) Å	*T* = 298 K
β = 96.684 (3)°	Block, colorless
*V* = 1365.8 (8) Å^3^	0.23 × 0.23 × 0.22 mm
*Z* = 2	

### Data collection

**Table:**

Bruker SMART CCD area-detector diffractometer	2969 independent reflections
Radiation source: fine-focus sealed tube	2254 reflections with *I* > 2σ(*I*)
graphite	*R*_int_ = 0.039
ω scans	θ_max_ = 27.0°, θ_min_ = 2.3°
Absorption correction: multi-scan (*SADABS*; Sheldrick, 1996)	*h* = −12→12
*T*_min_ = 0.682, *T*_max_ = 0.692	*k* = −10→10
10859 measured reflections	*l* = −20→21

### Refinement

**Table:**

Refinement on *F*^2^	Primary atom site location: structure-invariant direct methods
Least-squares matrix: full	Secondary atom site location: difference Fourier map
*R*[*F*^2^ > 2σ(*F*^2^)] = 0.033	Hydrogen site location: inferred from neighbouring sites
*wR*(*F*^2^) = 0.074	H-atom parameters constrained
*S* = 1.05	*w* = 1/[σ^2^(*F*_o_^2^) + (0.029*P*)^2^ + 0.3081*P*] where *P* = (*F*_o_^2^ + 2*F*_c_^2^)/3
2969 reflections	(Δ/σ)_max_ < 0.001
191 parameters	Δρ_max_ = 0.32 e Å^−^^3^
0 restraints	Δρ_min_ = −0.29 e Å^−^^3^

### Special details

**Table:**

Geometry. All e.s.d.'s (except the e.s.d. in the dihedral angle between two l.s. planes) are estimated using the full covariance matrix. The cell e.s.d.'s are taken into account individually in the estimation of e.s.d.'s in distances, angles and torsion angles; correlations between e.s.d.'s in cell parameters are only used when they are defined by crystal symmetry. An approximate (isotropic) treatment of cell e.s.d.'s is used for estimating e.s.d.'s involving l.s. planes.
Refinement. Refinement of *F*^2^ against ALL reflections. The weighted *R*-factor *wR* and goodness of fit *S* are based on *F*^2^, conventional *R*-factors *R* are based on *F*, with *F* set to zero for negative *F*^2^. The threshold expression of *F*^2^ > σ(*F*^2^) is used only for calculating *R*-factors(gt) *etc*. and is not relevant to the choice of reflections for refinement. *R*-factors based on *F*^2^ are statistically about twice as large as those based on *F*, and *R*- factors based on ALL data will be even larger.

### Fractional atomic coordinates and isotropic or equivalent isotropic displacement parameters (Å^2^)

**Table:**

	*x*	*y*	*z*	*U*_iso_*/*U*_eq_	
Zn1	−0.00948 (3)	0.68795 (3)	−0.001959 (16)	0.03019 (10)	
N1	0.0009 (2)	0.7029 (2)	0.12421 (12)	0.0326 (5)	
N2	−0.1532 (2)	0.8702 (2)	0.01204 (12)	0.0340 (5)	
N3	0.0351 (2)	0.7651 (3)	−0.10817 (13)	0.0431 (6)	
N4	0.1167 (2)	0.7042 (3)	−0.14488 (13)	0.0392 (5)	
N5	0.1955 (3)	0.6510 (3)	−0.18282 (16)	0.0595 (7)	
O1	0.13472 (15)	0.5071 (2)	0.02040 (9)	0.0301 (4)	
C1	0.2225 (2)	0.5932 (3)	0.15413 (14)	0.0334 (6)	
C2	0.2369 (2)	0.5228 (3)	0.07861 (14)	0.0298 (6)	
C3	0.3638 (3)	0.4688 (3)	0.06437 (17)	0.0406 (7)	
H3	0.3737	0.4146	0.0166	0.049*	
C4	0.4749 (3)	0.4940 (4)	0.11971 (18)	0.0480 (7)	
H4	0.5588	0.4596	0.1082	0.058*	
C5	0.4617 (3)	0.5702 (4)	0.19199 (19)	0.0525 (8)	
H5	0.5369	0.5906	0.2285	0.063*	
C6	0.3372 (3)	0.6157 (3)	0.20958 (16)	0.0442 (7)	
H6	0.3284	0.6625	0.2593	0.053*	
C7	0.0908 (3)	0.6441 (3)	0.17739 (15)	0.0342 (6)	
C8	0.0687 (3)	0.6214 (4)	0.26424 (16)	0.0560 (8)	
H8A	0.1082	0.7113	0.2955	0.084*	
H8B	0.1095	0.5208	0.2840	0.084*	
H8C	−0.0256	0.6177	0.2685	0.084*	
C9	−0.1276 (3)	0.7576 (4)	0.14743 (16)	0.0431 (7)	
H9A	−0.1132	0.8136	0.1988	0.052*	
H9B	−0.1844	0.6634	0.1534	0.052*	
C10	−0.1962 (2)	0.8714 (3)	0.08491 (16)	0.0355 (6)	
C11	−0.2991 (3)	0.9726 (3)	0.10270 (18)	0.0459 (7)	
H11	−0.3287	0.9701	0.1533	0.055*	
C12	−0.3567 (3)	1.0770 (4)	0.0441 (2)	0.0506 (8)	
H12	−0.4257	1.1465	0.0547	0.061*	
C13	−0.3116 (3)	1.0777 (4)	−0.03019 (19)	0.0473 (7)	
H13	−0.3491	1.1481	−0.0703	0.057*	
C14	−0.2102 (3)	0.9730 (3)	−0.04432 (17)	0.0396 (7)	
H14	−0.1800	0.9733	−0.0948	0.048*	

### Atomic displacement parameters (Å^2^)

**Table:**

	*U*^11^	*U*^22^	*U*^33^	*U*^12^	*U*^13^	*U*^23^
Zn1	0.03556 (18)	0.03175 (17)	0.02340 (16)	0.00139 (13)	0.00400 (11)	−0.00148 (14)
N1	0.0386 (12)	0.0332 (12)	0.0262 (11)	0.0014 (9)	0.0043 (9)	−0.0025 (9)
N2	0.0413 (12)	0.0299 (11)	0.0308 (12)	0.0010 (9)	0.0041 (10)	−0.0026 (9)
N3	0.0470 (14)	0.0501 (15)	0.0344 (13)	0.0087 (11)	0.0146 (11)	0.0093 (11)
N4	0.0450 (13)	0.0409 (14)	0.0319 (13)	−0.0022 (11)	0.0045 (10)	0.0062 (11)
N5	0.0658 (17)	0.0638 (18)	0.0530 (17)	0.0110 (14)	0.0245 (14)	−0.0008 (14)
O1	0.0310 (9)	0.0325 (9)	0.0262 (8)	−0.0003 (7)	0.0005 (7)	−0.0032 (8)
C1	0.0381 (14)	0.0353 (15)	0.0260 (14)	−0.0031 (12)	0.0006 (11)	0.0024 (11)
C2	0.0314 (13)	0.0274 (14)	0.0301 (13)	−0.0021 (10)	0.0015 (10)	0.0040 (10)
C3	0.0392 (15)	0.0424 (17)	0.0402 (16)	0.0043 (12)	0.0049 (12)	−0.0018 (12)
C4	0.0339 (15)	0.0534 (18)	0.0560 (19)	0.0030 (14)	0.0016 (13)	0.0051 (16)
C5	0.0433 (17)	0.0581 (19)	0.051 (2)	−0.0048 (15)	−0.0143 (15)	0.0006 (16)
C6	0.0506 (17)	0.0463 (17)	0.0332 (15)	−0.0013 (14)	−0.0053 (13)	−0.0014 (13)
C7	0.0453 (15)	0.0340 (14)	0.0238 (13)	−0.0013 (12)	0.0053 (11)	−0.0027 (11)
C8	0.068 (2)	0.072 (2)	0.0289 (16)	0.0102 (17)	0.0078 (15)	0.0062 (15)
C9	0.0452 (16)	0.0507 (17)	0.0359 (16)	0.0090 (13)	0.0150 (13)	0.0021 (13)
C10	0.0360 (14)	0.0336 (14)	0.0376 (16)	−0.0019 (11)	0.0077 (12)	−0.0054 (12)
C11	0.0395 (16)	0.0498 (19)	0.0503 (18)	0.0043 (13)	0.0136 (13)	−0.0045 (14)
C12	0.0388 (16)	0.0424 (17)	0.071 (2)	0.0083 (14)	0.0086 (15)	−0.0101 (16)
C13	0.0469 (17)	0.0376 (16)	0.056 (2)	0.0049 (14)	−0.0023 (15)	0.0045 (15)
C14	0.0440 (16)	0.0383 (17)	0.0364 (15)	−0.0016 (12)	0.0036 (12)	0.0003 (12)

### Geometric parameters (Å, °)

**Table:**

Zn1—N3	1.986 (2)	C4—C5	1.381 (4)
Zn1—O1^i^	2.0321 (17)	C4—H4	0.9300
Zn1—O1	2.0733 (17)	C5—C6	1.371 (4)
Zn1—N1	2.106 (2)	C5—H5	0.9300
Zn1—N2	2.107 (2)	C6—H6	0.9300
Zn1—Zn1^i^	3.0763 (11)	C7—C8	1.508 (3)
N1—C7	1.286 (3)	C8—H8A	0.9600
N1—C9	1.463 (3)	C8—H8B	0.9600
N2—C10	1.341 (3)	C8—H8C	0.9600
N2—C14	1.341 (3)	C9—C10	1.506 (4)
N3—N4	1.190 (3)	C9—H9A	0.9700
N4—N5	1.157 (3)	C9—H9B	0.9700
O1—C2	1.338 (3)	C10—C11	1.384 (3)
O1—Zn1^i^	2.0321 (17)	C11—C12	1.376 (4)
C1—C6	1.407 (3)	C11—H11	0.9300
C1—C2	1.412 (3)	C12—C13	1.372 (4)
C1—C7	1.482 (3)	C12—H12	0.9300
C2—C3	1.396 (3)	C13—C14	1.372 (4)
C3—C4	1.382 (4)	C13—H13	0.9300
C3—H3	0.9300	C14—H14	0.9300
			
N3—Zn1—O1^i^	108.26 (9)	C3—C4—H4	119.9
N3—Zn1—O1	99.31 (8)	C6—C5—C4	119.6 (3)
O1^i^—Zn1—O1	82.94 (7)	C6—C5—H5	120.2
N3—Zn1—N1	152.79 (9)	C4—C5—H5	120.2
O1^i^—Zn1—N1	98.94 (7)	C5—C6—C1	121.5 (3)
O1—Zn1—N1	84.73 (7)	C5—C6—H6	119.2
N3—Zn1—N2	96.00 (9)	C1—C6—H6	119.2
O1^i^—Zn1—N2	98.60 (8)	N1—C7—C1	120.0 (2)
O1—Zn1—N2	163.29 (7)	N1—C7—C8	122.9 (2)
N1—Zn1—N2	78.59 (8)	C1—C7—C8	117.1 (2)
N3—Zn1—Zn1^i^	108.42 (7)	C7—C8—H8A	109.5
O1^i^—Zn1—Zn1^i^	41.98 (5)	C7—C8—H8B	109.5
O1—Zn1—Zn1^i^	40.96 (5)	H8A—C8—H8B	109.5
N1—Zn1—Zn1^i^	92.33 (6)	C7—C8—H8C	109.5
N2—Zn1—Zn1^i^	138.10 (6)	H8A—C8—H8C	109.5
C7—N1—C9	120.1 (2)	H8B—C8—H8C	109.5
C7—N1—Zn1	128.44 (17)	N1—C9—C10	110.6 (2)
C9—N1—Zn1	109.99 (15)	N1—C9—H9A	109.5
C10—N2—C14	118.6 (2)	C10—C9—H9A	109.5
C10—N2—Zn1	113.83 (17)	N1—C9—H9B	109.5
C14—N2—Zn1	127.36 (18)	C10—C9—H9B	109.5
N4—N3—Zn1	124.78 (19)	H9A—C9—H9B	108.1
N5—N4—N3	176.9 (3)	N2—C10—C11	121.9 (3)
C2—O1—Zn1^i^	126.58 (14)	N2—C10—C9	117.3 (2)
C2—O1—Zn1	121.70 (14)	C11—C10—C9	120.8 (2)
Zn1^i^—O1—Zn1	97.06 (7)	C12—C11—C10	118.7 (3)
C6—C1—C2	118.8 (2)	C12—C11—H11	120.7
C6—C1—C7	118.7 (2)	C10—C11—H11	120.7
C2—C1—C7	122.5 (2)	C13—C12—C11	119.5 (3)
O1—C2—C3	119.0 (2)	C13—C12—H12	120.2
O1—C2—C1	122.7 (2)	C11—C12—H12	120.2
C3—C2—C1	118.2 (2)	C14—C13—C12	118.9 (3)
C4—C3—C2	121.5 (3)	C14—C13—H13	120.5
C4—C3—H3	119.3	C12—C13—H13	120.5
C2—C3—H3	119.3	N2—C14—C13	122.3 (3)
C5—C4—C3	120.1 (3)	N2—C14—H14	118.8
C5—C4—H4	119.9	C13—C14—H14	118.8

Symmetry codes: (i) −*x*, −*y*+1, −*z*.
